# Supplementary material for: Integration and mining of malaria molecular, functional and pharmacological data: how far are we from a chemogenomic knowledge space?
Source: Malar J. 2006 Nov 17;5:110. doi: 10.1186/1475-2875-5-110 (PMC1665468; doi:10.1186/1475-2875-5-110)
Supplement: Additional File 1 — Non-redundant malarial structures in the Protein Data Bank (PDB). The table compiles non redundant entries for Plasmodium protein structures in the Protein Data Bank at the date of writing. [file 1475-2875-5-110-S1.doc]

## Additional file 1 - Non-redundant malarial structures in the Protein Data Bank (PDB).

| **Description** | **PDB Ids** | **Ligands** | **Species** |
| --- | --- | --- | --- |
| 1-Cys peroxiredoxin | 1XIY, 1XCC |  | *P. f.*, *P. y.* |
| 2-Cys peroxiredoxin | 2H66, 2FEG |  | *P. v.* |
| 6-pyruvoyl tetrahydropterin synthase | 1Y13, 2AOS | Biopterin | *P. f.*, *P. v.* |
| Acyl CoA binding protein | 1HBK |  | *P. f.* |
| Adenylosuccinate synthetase | 1P98 |  | *P. f.* |
| Adenylosuccinate lyase | 2HVG |  | *P. v.* |
| Apical membrane antigen 1 | 1HN6, 1Z40, 1W81, 1W8K |  | *P. f.*, *P. v.* |
| beta-hydroxyacyl-ACP dehydratase | 1Z6B, 1ZHG | Cacodylate | *P. f.* |
| Cell division control protein 2 homolog, Pfpk5 | 1V0P, 1VOB, 1V0O, 1OB3 | Purvalanol B, Indirubin-5-Sulphonate | *P. f.* |
| ClpP protease | 2F6I |  | *P. f.* |
| C-terminal merozoite surface protein | 1B9W |  | *P. c.* |
| cyclophilin, peptidyl-prolyl cis-trans isomerase | 1QNH, 1QNG, 1Z81, 2B71, 2FU0 | Cyclosporin A | *P. f.*, *P. y.* |
| DHFR-TS | 1J3J, 1J3I, 1J3K, 2BL9, 2BLB | Pyrimethamine, NADPH , dUMP, WR99210, NDP, CP6, MES, CP7 | *P. f.*, *P. v.* |
| Dihydroorotate dehydrogenase | 1TV5 | orotic acid, n8e, fmn, a26 | *P. f.* |
| Dimethyladenosine transferase | 2H1R |  | *P. f.* |
| dUTPase | 1VYQ | 2 ,3-deoxy-3-fluoro-5-o-trityluridine | *P. f.* |
| Dynein Light Chain 1 | 1YO3 |  | *P. f.* |
| Enoyl-acyl-carrier protein reductase | 1NHG, 1NHW, 1NHD/ 1VRW, 1NNU | Triclosan, NAD+, TCC, TCT | *P. f.* |
| Falcipain-2 | 1YVB, 2GHU |  | *P. f.* |
| Ferredoxin | 1IUE |  | *P. f.* |
| Fe-superoxide dismutase | 2AWP, 2A03 |  | *P. k.*, *P. b.* |
| Fructose-bisphosphate aldolase | 1A5C |  | *P. f.* |
| Glutamate dehydrogenase | 2BMA |  | *P. f.* |
| Glutathione reductase | 1ONF |  | *P. f.* |
| Glutathione S-transferase | 1PA3, 1Q4J, 1OKT, 2AAW | S-Hexyl-Gsh, P33, GTX, DTL | *P. f.* |
| Glyceraldehyde-3-phosphate dehydrogenase | 1YWG, 2B4R, 2B4T | NAD+, glycerol, AES | *P. f.* |
| Glycerol-3-phosphate dehydrogenase | 1YJ8 |  | *P. f.* |
| Guanylate kinase | 1Z6G | 4-(2-hydroxyethyl)-1-piperazine ethanesulfonic acid | *P. f.* |
| Hypothetical protein | 1ZSO |  | *P. f.* |
| Hypoxanthine phosphoribosyl-transferase | 1CJB |  | *P. f.* |
| L-lactate dehydrogenase | 1LDG, 1CET, 1CEQ, 1T26, 1T2E, 1T25, 1T24, 1T2D, 1T2C, 1U4O, 1U5S, 1U5C, 1U5A, 1XIV, 2A94, 1OC4, 2A92, 2AA3 | NADH,Oxamate, Chloroquine, 4-Hydroxy-1,2,5-Thiadiazole-3-Carboxylic Acid, 3-Hydroxyisoxazole-4-Carboxylic Acid, NAD+, 4-Hydroxy-1,2,5-Oxadiazole-3-Carboxylic Acid, 2,6-dicarboxynaphthalene, naphthalene-2,6-disulfonic acid, 3,7-dihydroxynaphthalene-2-carboxylic acid, RB2, glycerol, Acetyl pyridine adenine dinucleotide, 1,4-dihydronicotinamide adenine dinucleotide, ADAPH | *P. f.*, *P. b.*, *P. v.* |
| MDR 2 | 2GHI |  | *P. y.* |
| MSP1 | 1OB1, 2FLG, 1CEJ, 1N1I |  | *P. f.*, *P. k.* |
| MSP3 | 1PSM |  | *P. f.* |
| MTIP-MyoA complex | 2AUC |  | *P. k.* |
| Nucleoside diphosphate kinase B | 1XIQ |  | *P. f.* |
| Ornithine aminotransferase | 1Z7D |  | *P. y.* |
| Oxoacyl-ACP reductase | 2C07 |  | *P. f.* |
| Phosphatidylethanolamine-binding protein | 2GZQ |  | *P. v.* |
| Phosphoglycerate kinase | 1LTK |  | *P. f.* |
| Phosphoglycerate mutase | 1XQ9 | SCN | *P. f.* |
| Plasmepsin | 1LS5, 1SME, 1ME6, 1LF2, 1LF4, 1PFZ, 1LEE, 1LF3, 1M43, 1MIQ, 2ANL, 1QS8, 1W6H, 1W6I, 2BJU, 1XE5, 1XE6, 1XDH | Peptstatin A, Inhibitor Rs370, Statine analog, Inhibitor Rs367, Inhibitor Eh58, JE2 | *P. f.*, *P. v.*, *P. m.* |
| *P. f.* gamete antigen 27/25 | 1N81 |  | *P. f.* |
| Purine nucleoside phosphorylase | 2BSX | Inosine | *P. f.* |
| Putative adenosine deaminase | 2AMX |  | *P. y.* |
| Putative deoxyribose-phosphate aldolase | 2A4A |  | *P. y.* |
| Putative, dim1 protein homolog | 2AV4 |  | *P. y.* |
| putative FK506-binding protein PFL2275c | 2FBN |  | *P. f.* |
| Putative formylmethionine deformylase | 1JYM, 1RQC, 1RL4 | BRR, BL5 | *P. f.* |
| putative HAD/COF-like hydrolase | 2B30 |  | *P. v.* |
| Putative, heat shock protein | 1Y6Z |  | *P. f.* |
| Putative, histamine-releasing factor | 1TXJ |  | *P. k.* |
| Putative, orotidine-monophosphate-decarboxylase | 2AQW, 2FDS, 2GUU, 2FFC, UP6 | Sulphate, SeMethionine, Uridine-5'-monophosphate | *P. y.*, *P. b.*, *P. v.* |
| Putative uridine phosphorylase | 1Q1G, 1SQ6, 1NW4 | 5'- Methylthio-Immucillin-H, Immh | *P. f.* |
| Putative, vacuolar protein sorting 29 | 2BDD |  | *P. y.* |
| Pvs25 | 1Z1Y, 1Z27 |  | *P. v.* |
| Rab6 | 1D5C | GDP | *P. f.* |
| Ribose 5-phosphate isomerase | 2F8M | Phosphate | *P. f.* |
| Ribulose 5-phosphate 3-Epimerase | 1TQX |  | *P. f.* |
| Spermidine synthase | 2FEG | MTA | *P. f.* |
| Thioredoxin | 1SYR, 2AV4 |  | *P. f.*, *P. y.* |
| Thioredoxin peroxidase I | 2H01 |  | *P. y.* |
| Triose Phosphate Isomerase | 1LYX, 1LZO, 1M7O, 1O5X, 1VGA, 1WOA, 1WOB, 1YDV, 1M7P | 2-Phosphoglycolic acid, Glycerol-3-Phosphate, 2-Phosphoglycolic acid, 3-Phosphoglyceric acid, 2-phosphoglycerate | *P. f.* |
| Ubiquitin conjugating enzyme E2 | 2H2Y, 2FO3 |  | *P. f.*, *P. v.* |

All entries were compiled from the PDB, and were cross-checked against PlasmoDB ([196], versions 4 and 5), the Structural Genomics Consortium, [145] and the Structural Genomics of Pathogenic Protozoa, [146]). *P. b.*, *Plasmodium berghei*; *P. c.*, *Plasmodium cynomolgi*; *P.f.*, *Plasmodium falciparum*, *P. k.*, *Plasmodium knowlesii*; *P. m.*, *Plasmodium malariae*, *P. v.*, *Plasmodium vivax*
